# Electrospun Poly(γ–glutamic acid)/β–Tricalcium Phosphate Composite Fibrous Mats for Bone Regeneration

**DOI:** 10.3390/polym11020227

**Published:** 2019-02-01

**Authors:** Chun-Hsu Yao, Shau-Pei Yang, Yueh-Sheng Chen, Kuo-Yu Chen

**Affiliations:** 1 Biomaterials Translational Research Center, China Medical University Hospital, Taichung 40202, Taiwan; chyao@mail.cmu.edu.tw; 2 Department of Biomedical Imaging and Radiological Science, China Medical University, Taichung 40202, Taiwan; samsam172may69@gmail.com; 3 School of Chinese Medicine, China Medical University, Taichung 40402, Taiwan; yuehsc@mail.cmu.edu.tw; 4 Department of Bioinformatics and Medical Engineering, Asia University, Taichung 41354, Taiwan; 5 Department of Chemical and Materials Engineering, National Yunlin University of Science and Technology, Yunlin 64002, Taiwan

**Keywords:** poly(γ–glutamic acid), β–tricalcium phosphate, bone substitute, electrospinning

## Abstract

A poly(γ–glutamic acid)/β–tricalcium phosphate (γ–PGA/β–TCP) composite fibrous mat was fabricated using the electrospinning technique as a novel bone substitute. The mat was then cross-linked with cystamine in the presence of 1-ethyl-3-(3-dimethylaminopropyl)-carbodiimide to improve its water-resistant ability. Scanning electron micrographs revealed that the γ–PGA/β–TCP fibers had a uniform morphology with diameters ranging from 0.64 ± 0.07 µm to 1.65 ± 0.16 µm. The average diameter of the fibers increased with increasing cross-linking time. Moreover, increasing the cross-linking time and decreasing the γ–PGA/β–TCP weight ratio decreased the swelling ratio and in vitro degradation rate of the composite fibrous mat. In vitro experiments with osteoblast-like MG-63 cells demonstrated that the mat with a γ–PGA/β–TCP weight ratio of 20 and cross-linked time of 24 h had a higher alkaline phosphatase activity and better cell adhesion. Furthermore, the rat cranial bone defect was created and treated with the γ–PGA/β–TCP composite fibrous mat to evaluate its potential in bone regeneration. After 8 weeks of implantation, micro computed tomography showed that the γ–PGA/β–TCP composite fibrous mat promoted new bone growth. These observations suggest that the γ–PGA/β–TCP composite fibrous mat has a potential application in bone tissue engineering.

## 1. Introduction

The repair of bone defects remains a great challenge in orthopedic and maxillofacial surgery. Various materials have been used as bone substitutes to reconstruct bone defects caused by trauma, tumor removal, infections, or skeletal abnormalities. β–tricalcium phosphate (β–TCP), a bioactive and biodegradable ceramic, is commonly used in clinical settings to enhance the biocompatibility and osteoconductivity of bone replacements [1]. However, it is difficult to maintain in the reconstructed area and lacks structural stability. Poly(γ–glutamic acid) (γ–PGA), a naturally occurring anionic poly(amino acid), is secreted by several microorganisms such as the *Bacillus subtilis* strain [2]. γ–PGA is degraded in vivo by γ–glutamyl transpeptidase into glutamic acid, which is nontoxic and widely distributed in the human body [3]. Due to its good biocompatibility, biodegradability, and high water-retention capability, γ–PGA has been studied in biomedical applications such as drug delivery, bioadhesive, wound dressing, and tissue engineering [4,5,6,7]. A previous report has confirmed that γ–PGA could bind β–TCP to form composites with good cytocompatibility [8]. However, γ–PGA dissolves rather rapidly in aqueous environments, which reduces its utility as a scaffold in tissue repair. The use of a proper cross-linking agent to maintain the physical properties of γ–PGA under physiological conditions is desirable for biomedical applications. Cross-linking agents, such as ethyleneglycol diglycidylether [6,9], 2,2′-(ethylenedioxy) diethylamine [10], cystamine [11,12,13], and L-lysine [14] have been used to cross-link γ–PGA to improve its water stability. Of these, cystamine with disulfide bonds can cross-link γ–PGA in the presence of 1-ethyl-3-(3-dimethylaminopropyl)-carbodiimide (EDC) via the reaction between the amine groups of cystamine and the carboxyl groups of γ–PGA [13]. The disulfide-cross-linked γ–PGA is reduction-sensitive under physiological conditions because the cleavage of the disulfide-cross-linkage to thiol groups is controllable by using various reductants [11,12].

An ideal bone substitute should mimic the structure and physicochemical properties of the native bone extracellular matrix (ECM) to induce bone cell adhesion, proliferation, and differentiation [15]. The native ECM is composed of fibers, pores, and ridges in the submicron to nanometer range. Electrospinning is an attractive technique for fabricating nano- and microscale fibrous scaffolds from natural and synthetic materials for tissue engineering applications due to the simplicity and effectiveness of the process [16,17,18,19]. A number of natural and synthetic polymers have been electrospun to create fibrous scaffolds for bone tissue engineering applications, such as collagen, gelatin, chitosan, cellulose, poly(vinyl alcohol), poly(lactic acid), poly(lactide-*co*-glycolide), and poly(caprolactone) [20,21,22,23,24,25]. These fibrous scaffolds generated via electrospinning provide a high specific surface area with appropriate porosity and three-dimensional network structures to support cell attachment, migration, and proliferation. Moreover, several studies have demonstrated good cell adhesion and proliferation on the electrospun γ–PGA or γ–PGA-based composite fibrous mats [7,12,13,26,27].

Shu et al. [8] prepared porous γ–PGA/β–TCP composites through the solvent casting, particle leaching, and freezing-drying methods and found that MC3T3 cells proliferated well in the presence of γ–PGA/β–TCP extracts. To date, no study of the preparation of γ–PGA/β–TCP composite fibrous mats using the electrospinning method has been published. The current study fabricated biodegradable composite fibrous mats with different weight ratios of γ–PGA/β–TCP as novel bone substitutes by electrospinning. The mats were then cross-linked with cystamine at different times to render the electrospun fibers with good water stability. The effects of the γ–PGA/β–TCP weight ratio and cross-linking time on the morphology, water uptake capability, weight loss, cytotoxicity, and cellular response of the composite fibrous mats were investigated. The cellular response was assessed in vitro by a 3-(4,5-dimethylthiazolyl)-2,5-diphenyltetrazolium bromide (MTT) colorimetric assay of cell viability, an alkaline phosphatase (ALP) activity assay of osteoblast differentiation, and a scanning electron microscope (SEM) observation of cell morphology. Finally, the γ–PGA/β–TCP composite fibrous mats were implanted into the rat calvarial defects for 4 and 8 weeks to evaluate their effectiveness in bone repair in vivo. New bone formation was examined using a micro-computed tomography (micro-CT) system.

## 2. Materials and Methods

### 2.1. Preparation of Electrospun γ–PGA/β–TCP Composite Fibrous Mats

β–TCP nanoparticles (Shimakyu’s Pure Chemicals, Osaka, Japan) with an average particle size of 20–40 nm were dispersed in deionized water by ultrasonication. γ–PGA powders (Vedan Enterprise Co., Taichung, Taiwan) were then dissolved at a concentration of 20 wt % in aqueous β–TCP solution. The weight ratio of γ–PGA to β–TCP was 20:1 or 40:1. The mixed solution was loaded into a syringe with a metal needle (G22, diameter = 0.41 mm) and then electrospun onto an aluminum foil substrate using an electrostatic spinning apparatus (FES-COS, Falco Tech Enterprise Co., Ltd., New Taipei City, Taiwan) to produce γ–PGA/β–TCP composite fibrous mats. The typical electrospinning parameters were as follows: 20 kV (voltage), 13 cm (tip-to-collector distance), and 0.3 mL/h (feed rate). After electrospinning for 5 h, the γ–PGA/β–TCP composite fibrous mats were peeled off from the aluminum foil and cut into 1.5 cm × 1.5 cm squares. The mats were then immersed in 10 mg/mL EDC (Sigma-Aldrich, St. Louis, MO, USA) solution in ethanol to activate the carboxyl groups of the γ–PGA. After activation for 3 h, the mats were treated with 10 mg/mL cystamine (Sigma-Aldrich) solution in ethanol for 12 or 24 h to cross-link the carboxyl groups of the γ–PGA with the amine groups at the two ends of cystamine [12]. Finally, the cross-linked mats were rinsed with deionized water for 48 h to remove the residual cross-linking reagent, frozen at −80 °C, and then freeze-dried.

### 2.2. Fiber Morphology Observation

The morphology of the γ–PGA/β–TCP composite fiber was observed by SEM (Hitachi S-3000N, Tokyo, Japan). Before a SEM observation, the dry sample was sputter-coated with a layer of gold. The average diameter of the electrospun fibers was calculated by counting 50 individual fibers using the Image J software (National Institute of Health, Bethesda, MD, USA) from the SEM images.

### 2.3. Measuring Water Uptake Capability

The dry γ–PGA/β–TCP composite fibrous mat with a size of 1.5 cm × 1.5 cm was weighted (W_dry_) and then immersed in 10 mL of deionized water. After soaking for 3, 6, 12, 24, and 48 h at 37 °C, the swollen mat was taken out of the water, gently blotted with filter paper to remove the excess liquid, and immediately weighed (W_wet_). The water uptake percentage (ΔW%) at each period was determined according to the following equation: ΔW(%) = (W_wet_ − W_dry_)/W_dry_ × 100%.

### 2.4. Determination of In Vitro Degradation 

The dry composite fibrous mat with a dimension of 1.5 cm × 1.5 cm was weighed (Wi). Subsequently, the specimen was soaked in a phosphate buffer solution (PBS) at 37 °C. After soaking for 1, 2, 4, 6, and 8 weeks, the specimen was removed from the PBS, lyophilized, and weighed again (W_f_). The weight loss percentage (ΔW%) at each time point was calculated from the following formula: ΔW(%) = (W_i_ − W_f_)/W_i_ × 100%.

### 2.5. Cytotoxicity and Alkaline Phosphatase Activity Evaluation 

Following sterilization with ^60^Co gamma ray irradiation (10 kGy), each γ–PGA/β–TCP composite fibrous mat was placed in a sterilized tube filled with PBS. After soaking for 1, 2, 4, 6, and 8 weeks at 37 °C, the extracts were collected for cell culture. The osteoblast-like MG-63 human osteosarcoma cells (BCRC no. 60279, Bioresources Collection and Research Center, Hsichu, Taiwan) were seeded on a 48-well plate at a density of 1 × 10^4^ cells/well and incubated at 37 °C with 5% CO_2_. After culturing for 24 h, the growth medium (Eagle’s minimum essential medium (Gibco, Grand Island, NY, USA) supplemented with 10% fetal bovine serum (Gibco) and 1% penicillin–streptomycin solution (Gibco)) was replaced with a media containing extract liquid. In the control group, the culture medium was mixed with PBS. After 2 and 4 days of culture, the proliferation and differentiation of the osteoblasts were evaluated by MTT (USB, Amersham Life Science, Cleveland, OH, USA) assay and ALP activity assay, respectively. In brief, the medium was replaced with 10 μL/well of MTT solution (5 mg/mL) and 100 μL/well of culture medium. After incubating for 4 h at 37 °C in a 5% CO_2_ atmosphere, the solution was removed and dimethyl sulfoxide (Merck, Whitehouse Station, NJ, USA) was added and mixed thoroughly to dissolve the purple formazan crystals. The optical density of the formazan solution was measured using a plate reader (uQuant, Bio-Tek Instruments Inc., Sunnyvale, CA, USA) at a wavelength of 570 nm against a reference wavelength of 650 nm. For the analysis of ALP activity, the medium was replaced with a 20 μL/well of 0.1% Triton X-100 (Sigma-Aldrich) and incubated at room temperature for 5 min for cell lysis. Then added was 100 μL/well of the commercially available ALP assay kit (Procedure No. DG1245-K; Sigma-Aldrich) to produce p-nitrophenol. The absorbance at 405 nm caused by the production of p-nitrophenol was assessed for 30 min using an ELISA reader. The change in the rate of absorbance was directly proportional to the ALP activity.

### 2.6. MG-63 Cells Cultured with the Fibrous Mat 

The effect of MG-63 cells on the γ–PGA/β–TCP composite fibrous mat was investigated to evaluate directly the cytocompatibility of such a material. After it had been sterilized with ^60^Co gamma ray irradiation, the fibrous mat was placed in a 24-well culture dish, seeded with 1 × 10^4^ MG-63 cells, immersed in culture medium, and then incubated at 37 °C with 5% CO_2_ atmosphere for 6 h. At the end of incubation, the sample was washed with PBS to remove the non-adherent cells and then fixed with 2 vol% glutaraldehyde aqueous solution (Panreac, Barcelona, Spain) for 1 h. After being thoroughly washed with PBS, the cells were dehydrated in a graded series of ethanol solutions (30, 50, 60, 70, 80, 90, 95, and 100%) and then dried in a critical point drier (HCP-2, Hitachi, Tokyo, Japan). The dried sample was immediately sputter-coated with gold to observe the cellular morphology by SEM.

### 2.7. In Vivo Biological Response Evaluation

The experimental cranial implantation was conducted on adult Sprague–Dawley rats (300–350 g, National Laboratory Animal Center, Taipei, Taiwan). The Ethical Committee for Animal Experiments at China Medical University, Taichung, Taiwan had approved the protocols. The rats were anesthetized with an intramuscular injection of Zoletil 50 (Virbac, Carros, France) and 2% Rompun solution (Bayer, Leverkusen, Germany) (1:2 ratio, 0.4 mL/kg). The hair on the rat cranium was shaved and disinfected with 70% ethanol and 10% povidone–iodine solution (Chou Jen Pharmaceutical Co., Nantou, Taiwan). The skull was exposed with a midline scalp incision, and the overlying parietal pericranium was then cut. A high-speed oscillating saw was used to generate a circular defect of the parietal bone with a diameter of 8 mm. The sterile γ–PGA/β–TCP composite fibrous mat with a circular shape and a diameter of 8 mm was implanted into the calvarial bone defect to assess its bone healing capability in vivo. The defect without implantation was used as a negative control. The anesthetized rats were sacrificed by administering an overdose of sodium pentobarbital at 4 and 8 weeks post-operatively. The crania were removed, washed with PBS, and fixed in a 10% neutral formalin-buffered solution (Merck) for 24 h. The repair of the bone defect was observed by micro-CT (SkyScan 1176, Bruker, Kontich, Belgium). The regenerated bone was quantified by counting the number of bone voxels using Image J software and was expressed as a percentage of the ingrown bone tissue in the created bone defect.

### 2.8. Statistical Analysis 

All quantitative data were presented as mean ± standard deviation. Statistical analysis was performed using Student’s *t*-test or one-way analysis of variance (ANOVA), followed by post hoc Fisher’s least significant difference test. A difference was regarded as significant at *p* < 0.05.

## 3. Results and Discussion

### 3.1. Morphology of Electrospun γ–PGA/β–TCP Composite Fibers

**Figure 1:**
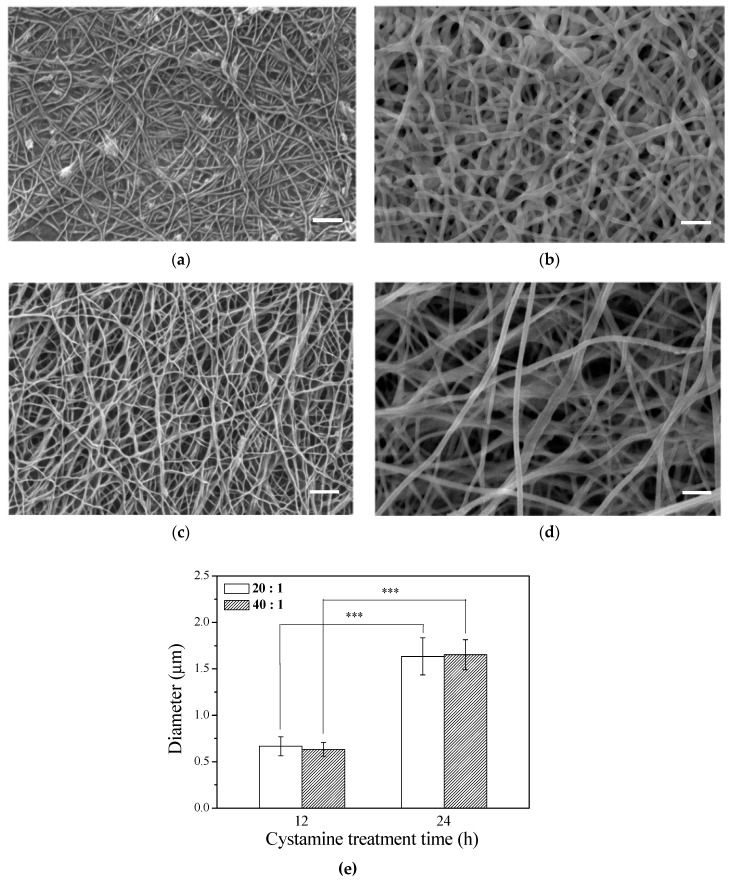
SEM images of the γ–PGA/β–TCP composite fibrous mats with different γ–PGA/β–TCP weight ratios and different cystamine treatment times: (**a**) 20:1, 12 h; (**b**) 20:1, 24 h; (**c**) 40:1, 12 h; and (**d**) 40:1, 24 h. (**e**) The diameter of the electrospun γ–PGA/β–TCP composite fibers (*** *p* < 0.005). Scale bars: 2 μm.

The microstructure of the scaffold has a profound influence on cell behavior. Electrospun fibers have been reported to be able to promote migration, cell adhesion, and proliferation. After the γ–PGA/β–TCP composite fibers were cross-linked with cystamine for 12 or 24 h, the surface morphology of the fibrous mats was observed by SEM (Figure 1a–d). The SEM micrographs of the γ–PGA/β–TCP fibrous mats prepared under optimized conditions exhibit continuous and uniform fibrous structure. The fibrous mat with a γ–PGA/β–TCP weight ratio of 40 (Figure 1c,d) had a larger pore size than that with a γ–PGA/β–TCP weight ratio of 20 (Figure 1a,b). Moreover, the pore size increased with increasing cystamine cross-linking time from 12 to 24 h. Additionally, the average diameter of the γ–PGA/β–TCP composite fibers also increased with increasing cystamine cross-linking time (Figure 1e), which probably resulted from the water absorption during the cross-linking process. Our previous study also showed that the average diameter of the gelatin fibers cross-linked with glutaraldehyde vapor increased with increasing cross-linking time [28]. However, the γ–PGA/β–TCP weight ratio had no significant effect on the diameter of the electrospun fibers.

### 3.2. Water Uptake Capability and In Vitro Degradation

**Figure 2:**
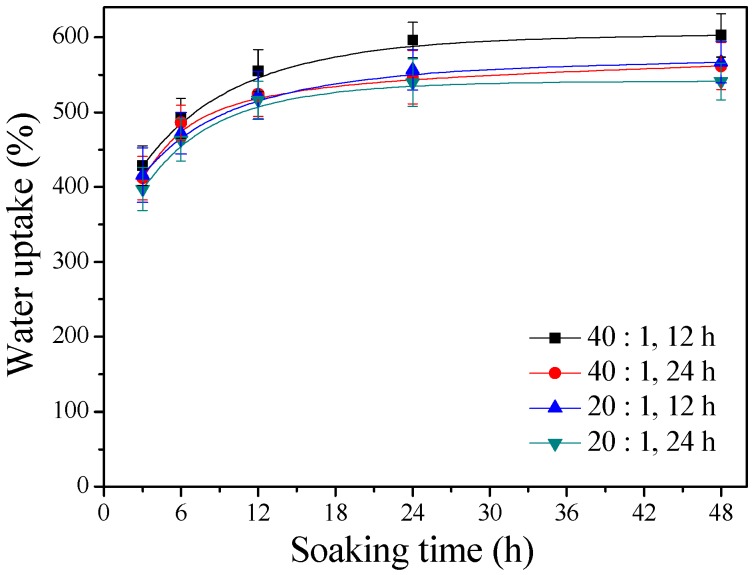
The water uptake capabilities of the γ–PGA/β–TCP composite fibrous mats.

Figure 2 displays the water uptake capabilities of the γ–PGA/β–TCP composite fibrous mats with different γ–PGA/β–TCP weight ratios and different cystamine treatment times. It was found that the water absorption increased with immersion time. The fibrous mats in water began to swell rapidly in 3 h but thereafter exhibited swelling at a significantly reduced rate (*p* < 0.005). Furthermore, the fibrous mats became saturated with water when the soaking period exceeded 24 h. Figure 2 also reveals that the water contents of the γ–PGA/β–TCP composite fibrous mats increased with increasing γ–PGA/β–TCP weight ratio and decreased as the cystamine treatment time increased. After 48 h of soaking, the γ–PGA/β–TCP composite fibrous mat prepared with a γ–PGA/β–TCP weight ratio of 40 and cross-linked with cystamine for 12 h had the highest water absorption value (602.6 ± 28.8%), while that obtained with a γ–PGA/β–TCP weight ratio of 20 and a cross-linking time of 24 h had the lowest water uptake (541.1 ± 24.5%). Shu et al. [8] also found that the swelling ratio of porous γ–PGA/β–TCP composite increased by increasing the percentage of γ–PGA. The high water-holding capacity of the γ–PGA/β–TCP composite fibrous mat was attributed to the hydrophilic nature of γ–PGA and to the high surface area-to-volume ratio and high porosity of the fibrous structures. Moreover, the water absorption behavior of the composite fibrous mat seemed to depend on the number of hydrophilic free carboxylate groups present. 

**Figure 3:**
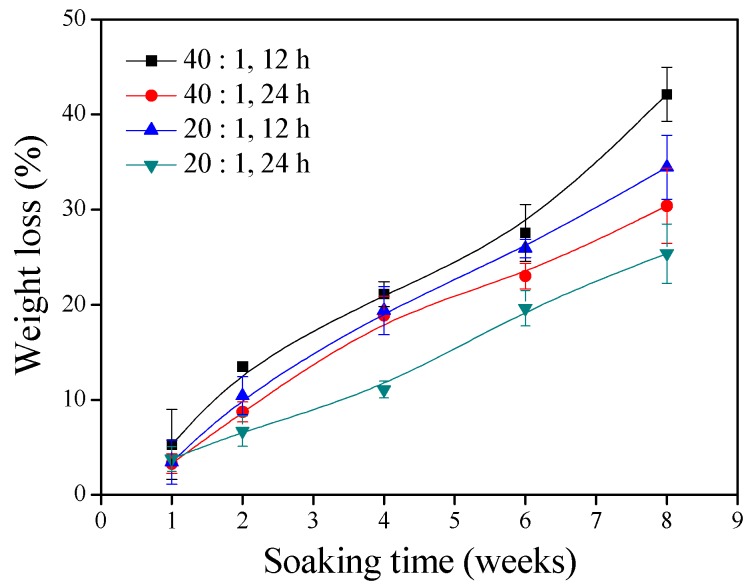
The weight losses of the γ–PGA/β–TCP composite fibrous mats during the soaking time.

In vitro hydrolytic degradation of the γ–PGA/β–TCP composite fibrous mats with different weight ratios of γ–PGA to β–TCP and different cystamine treatment times continued for 8 weeks (Figure 3). It was observed that the extent of degradation in the in vitro physiological environment increased with soaking time. The extent of degradation was significantly attenuated by decreasing the γ–PGA/β–TCP weight ratio and increasing the cystamine treatment time. The tendency of this result is well in agreement with the tendency of water absorption: the higher the water absorption, the lower the weight loss. The percentage weight remaining of the γ–PGA/β–TCP composite fibrous mats prepared with a γ–PGA/β–TCP weight ratio of 20 and cross-linked with cystamine for 24 h declined to 74.6% at week 8. These composite fibrous mats were further analyzed for their cellular toxicity.

### 3.3. Cell Viability and Alkaline Phosphatase Activity

**Figure 4:**
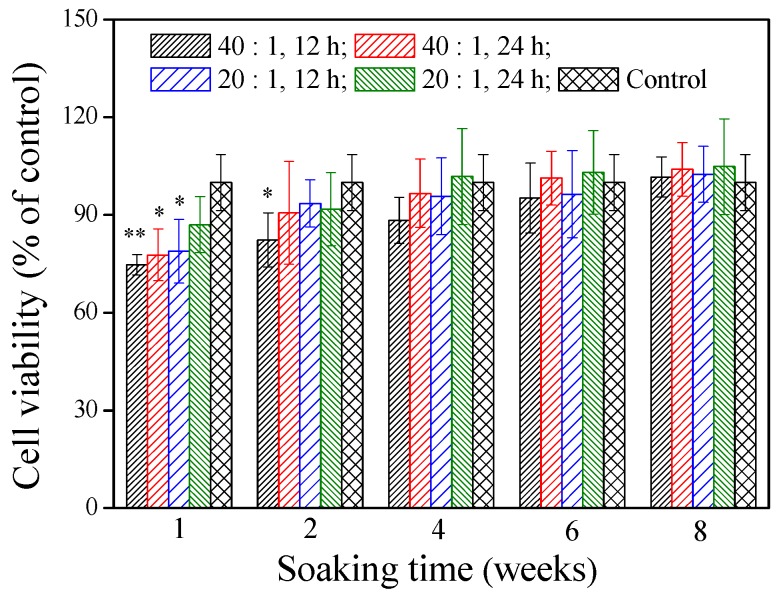
The effect of the γ–PGA/β–TCP composite fiber extract on the MG-63 cell viability by a MTT assay after 2 days of culture. The results are expressed as a percentage of the control (* *p* < 0.05 and ** *p* < 0.01 vs. control).

The cytotoxicity of the materials is very important for their potential use in vivo. The cell viability of the γ–PGA/β–TCP composite fibrous mat was assessed by the MTT assay after treatment of the MG-63 cells with the mat extracts for 2 days. Figure 4 shows that the extracts, except for the mat obtained with a γ–PGA/β–TCP weight ratio of 20 and cross-linked with cystamine for 24 h, had significantly less cells than the control group when the soaking period was 1 week (*p* < 0.05). Notably, the extract from the mat prepared with a γ–PGA/β–TCP weight ratio of 40 and cross-linked with cystamine for 12 h decreased the cell number most markedly (*p* < 0.01), in which the number of MG-63 cells was 25.3% lower than that in the control group. The inhibition of cell expansion suggests some cytotoxic effect of the residual EDC on the cells [29,30]. When the soaking period was 2 weeks, only the mat fabricated with a γ–PGA/β–TCP weight ratio of 40 and cross-linked with cystamine for 12 h had significantly less cells than the control group (*p* < 0.05). However, no remarkable difference in cell viability was found between the control group and the γ–PGA/β–TCP composite fibrous mat when the soaking period exceeded 4 weeks (*p* > 0.05). These findings suggest that the residues released from the γ–PGA/β–TCP composite fibrous mat were not harmless to MG-63 cells in vitro after 4 weeks of soaking.

**Figure 5:**
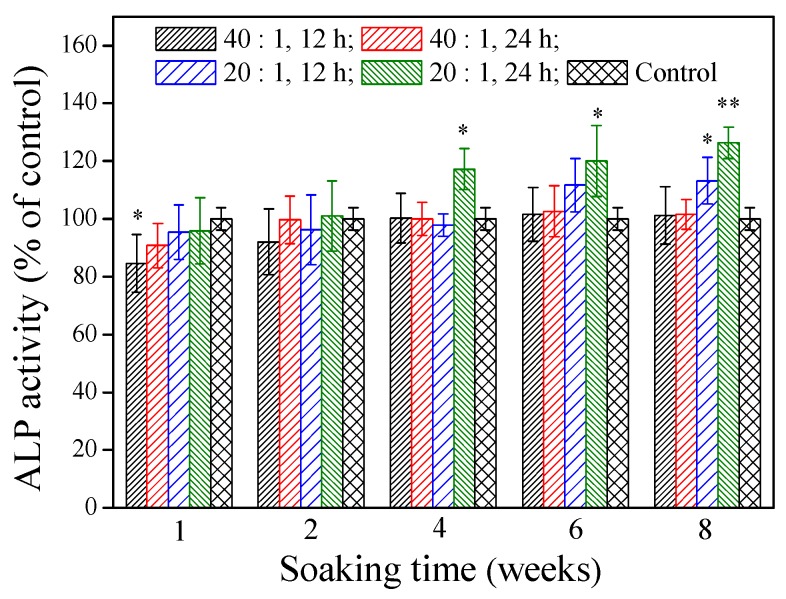
The effect of the γ–PGA/β–TCP composite fiber extract on the ALP activity of MG-63 cells after 4 days of culture. The results are expressed as a percentage of the control (* *p* < 0.05 and ** *p* < 0.01 vs. control).

ALP localized on the cell membrane of osteogenic cells is a differentiation marker of early osteoblasts. Differentiated osteoblasts exhibit high ALP activity. Figure 5 illustrates that various γ–PGA/β–TCP composite fibrous mats had different effects on the ALP activity of MG-63 cells after 4 days of culture. At a soaking time of 1 week, only the mat fabricated with a γ–PGA/β–TCP weight ratio of 40 and cross-linked with cystamine for 12 h had a significantly lower ALP activity than the control group (*p* < 0.05). No statistically significant difference in the ALP activity was observed between the control group and the γ–PGA/β–TCP composite fibrous mat when the soaking period was 2 weeks (*p* > 0.05). The extracts from the mat prepared with a γ–PGA/β–TCP weight ratio of 20 and cross-linked with cystamine for 24 h had a significantly higher ALP activity than the control group when the soaking period exceeded 4 weeks (*p* < 0.05). It increased the ALP activity by 17.3%, 20.1%, and 26.3% when the soaking period was 4, 6, and 8 weeks, respectively. Moreover, the mat with a γ–PGA/β–TCP weight ratio of 20 had a higher ALP activity than that with a γ–PGA/β–TCP weight ratio of 40 when the soaking period exceeded 6 weeks. These results suggest that the higher content of β–TCP enhances osteoblastic cell differentiation [31]. 

### 3.4. Cell Adhesion

**Figure 6:**
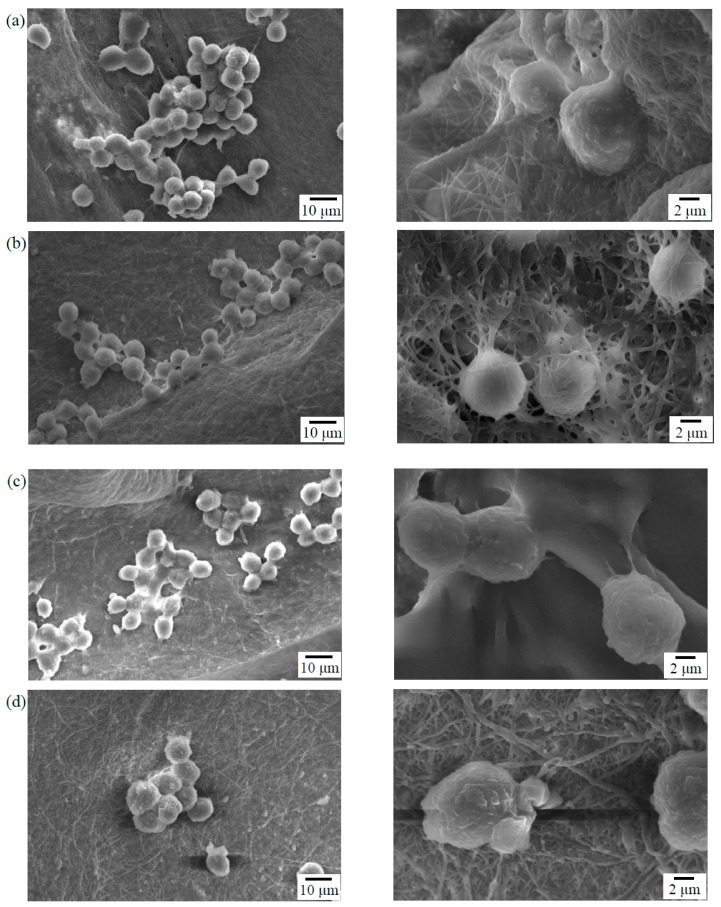
SEM photographs of the MG-63 cells that adhered to the surfaces of the γ–PGA/β–TCP composite fibrous mats with different γ–PGA/β–TCP weight ratios and different cystamine treatment times: (**a**) 20:1, 12 h; (**b**) 20:1, 24 h; (**c**) 40:1, 12 h; and (**d**) 40:1, 24 h after 6 h of culture.

Cells will undergo specific morphological changes to stabilize the cell-material interface after they contact the material surface. In vitro MG-63 cells adhesion experiments were performed to assess the cellular response on the electrospun γ–PGA/β–TCP fibers. Figure 6 presents the morphology of the MG-63 cells that were cultured on the surfaces of the γ–PGA/β–TCP fibrous mats with γ–PGA/β–TCP weight ratios of 20 and 40 and cross-linking times of 12 and 24 h. After 6 h of culture, the MG-63 cells attached well to the surfaces of the mat. The cell adhesion characteristic of the mats with a γ–PGA/β–TCP weight ratio of 20 was superior to that of the mats with a γ–PGA/β–TCP weight ratio of 40. The MG-63 cells that adhered to the mats with a γ–PGA/β–TCP weight ratio of 40 exhibited a round shape with short pseudopodia (Figure 6c,d). In contrast, the cells interacted well with the surrounding fibers having a γ–PGA/β–TCP weight ratio of 20 (Figure 6a,b). In particular, cell pseudopodia integration with the surrounding fibers was observed in the fibrous mat treated with cystamine for 24 h (Figure 6b). Moreover, the cells migrated into the fibrous network to form a 3-dimensional cell-scaffold construct, indicating it had better cytocompatibility. Duan et al. [32] also demonstrated that Jurkat cells could adhere to the fibers and grow inside the 3D electrospun polymer fiber sponges. Additionally, the SEM examination showed that the mats with a cross-linking time of 24 h had a more distinct fibrous morphology than those with a cross-linking time of 12 h, indicating that a longer cross-linking time could stabilize the fibrous structure of electrospun fibers throughout the culture period. The fine fibrous structure provided a good environment for cell adhesion. Because the electrospun mat with a γ–PGA/β–TCP weight ratio of 20 and cross-linking time of 24 h had a higher ALP activity and better cell adhesion, it was adopted in the follow-up animal study.

### 3.5. Biological Response of Rat Calvarial Bone 

The γ–PGA/β–TCP composite fibrous mats with a γ–PGA/β–TCP weight ratio of 20 and cross-linking time of 24 h were implanted into rat cranial bone defects. After implantation for 4 and 8 weeks, the repair of the calvarial bone defect was evaluated using the micro-CT. As a control, the bone defect without implantation was also analyzed. All animals survived until the experiment was terminated (8 weeks). The surgical incision healed rapidly without causing any wound infection, inflammation, scalp effusion, hematoma, festering, or other complications. 

**Figure 7:**
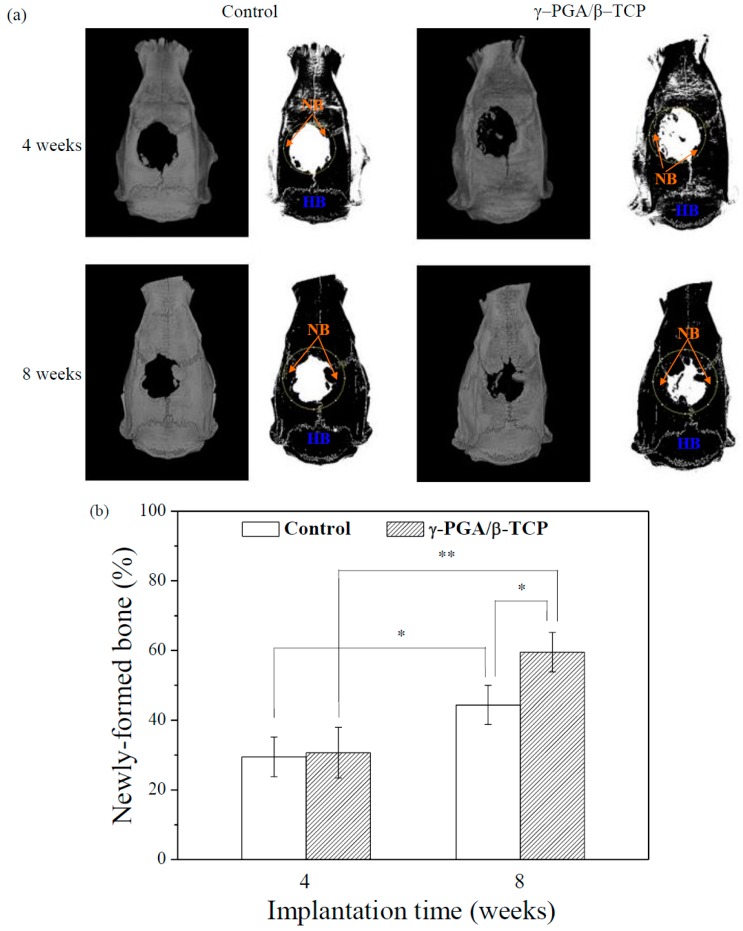
(**a**) Micro-CT images of the rat calvarial bones covered with or without γ–PGA/β–TCP composite fibrous mats obtained 4 and 8 weeks postimplantation (HB = host bone, NB = new bone). The dotted circles indicate the original defects. (**b**) The quantitative micro-CT analysis of the newly formed bones in the rat calvaria defects (* *p* < 0.05 and ** *p* < 0.01).

Figure 7a displays the micro-CT images of the 8 mm skull defects in the rats following the application of the γ–PGA/β–TCP composite fibrous mats. Figure 7b demonstrates the amount of new bone as a percentage of the volume of calvarial bone defect in each implantation period. At 4 weeks after surgery, the newly regenerated bone was observed at the periphery of the calvarial bone defect. The rounded bone defect became smaller and irregular in shape. However, the area of the newl formed bone in the defect treated with the γ–PGA/β–TCP composite fibrous mat was not significantly different from that obtained with the control (*p* > 0.05).

At 8 weeks post-operation, the area of the calvarial bone defect was significantly smaller than that found in the 4-week sample, indicating that the area of newly formed bone at the defect site increased with the implantation period. Furthermore, a significantly greater amount of new bone formation was observed for the defect treated with the γ–PGA/β–TCP composite fibrous mat as compared to the control group at 8 weeks of implantation (*p* < 0.05). Moreover, a notable increase in the percentage of bone repair from 30.7% to 59.5% at the sites treated with the γ–PGA/β–TCP composite fibrous mats from week 4 to week 8 was observed when compared to that from 29.5% to 44.4% at the sites without implant (control). 

An ideal bone replacement material should conduct cells to defect sites; induce bone cell adhesion, proliferation, and differentiation; and promote new bone formation. An electrospun fibrous mat could provide a high specific surface area for cell adhesion. In vitro MG-63 cell adhesion experiments showed that MG-63 cells attached well to the surfaces of the γ–PGA/β–TCP composite fibrous mat. Moreover, cells could integrate with the surrounding fibers and migrate into the fibrous network. Wang et al. [13] demonstrated that the three-dimensional structure of the γ–PGA nanofibers was favorable for L929 cell adhesion and migration. Gao et al. [26] also found that the filopodia of MC3T3-E1 cells tended to attach to and integrate with the surrounding fibers of the electrospun γ–PGA-silica hybrid scaffold. 

β–TCP, a biodegradable bone replacement material, is able to osteoconduct cells into the scaffold [33]. Moreover, the release of calcium ions from β–TCP could induce osteogenic differentiation of bone marrow stem cells [34]. In this study, the electrospun mat with a γ–PGA/β–TCP weight ratio of 20 and cross-linking time of 24 h had a higher ALP activity. Moreover, the ALP activity was increased with culture time. On the basis of these observations, the γ–PGA/β–TCP composite fibrous mat promoted new bone growth and could be used as a bone replacement material for damaged bone tissues.

## 4. Conclusions

In this investigation, γ–PGA/β–TCP composite fibers with γ–PGA/β–TCP weight ratios of 20 and 40 were successfully prepared via electrospinning method. The electrospun fibers were subsequently cross-linked with cystamine to make them more stable under physiological conditions. Decreasing the γ–PGA/β–TCP weight ratio and increasing the cystamine cross-linking time decreased the water uptake and weight loss of the γ–PGA/β–TCP composite fibrous mats. When the weight ratio of γ–PGA/β–TCP was 20 and the cross-linking time was 24 h, the composite fibrous mat had a higher ALP activity and better cell adhesion. Furthermore, the interactions between the cell pseudopodia and the surrounding fibers were clearly observed. Animal studies using the calvarial defect model revealed that the composite fibrous mat efficiently promoted bond defect repair after 8 weeks of implantation. Accordingly, the γ–PGA/β–TCP composite fibrous mat could be used as a bone substitute for bone tissue regeneration.
